# The Dilemmas of the Gourmet Fly: The Molecular and Neuronal Mechanisms of Feeding and Nutrient Decision Making in *Drosophila*

**DOI:** 10.3389/fnins.2013.00012

**Published:** 2013-02-12

**Authors:** Pavel M. Itskov, Carlos Ribeiro

**Affiliations:** ^1^Behaviour and Metabolism Laboratory, Champalimaud Neuroscience Programme, Champalimaud Centre for the UnknownLisbon, Portugal

**Keywords:** behavior, sensory systems, feeding, olfaction, taste, neuromodulators, neuropeptides, internal state

## Abstract

To survive and successfully reproduce animals need to maintain a balanced intake of nutrients and energy. The nervous system of insects has evolved multiple mechanisms to regulate feeding behavior. When animals are faced with the choice to feed, several decisions must be made: whether or not to eat, how much to eat, what to eat, and when to eat. Using *Drosophila melanogaster* substantial progress has been achieved in understanding the neuronal and molecular mechanisms controlling feeding decisions. These feeding decisions are implemented in the nervous system on multiple levels, from alterations in the sensitivity of peripheral sensory organs to the modulation of memory systems. This review discusses methodologies developed in order to study insect feeding, the effects of neuropeptides and neuromodulators on feeding behavior, behavioral evidence supporting the existence of internal energy sensors, neuronal and molecular mechanisms controlling protein intake, and finally the regulation of feeding by circadian rhythms and sleep. From the discussed data a conceptual framework starts to emerge which aims to explain the molecular and neuronal processes maintaining the stability of the internal milieu.

## Introduction

In order to survive and reproduce animals must provide themselves with an adequate supply of energy and nutrients. Under this selective pressure animals have evolved highly sophisticated and diverse repertoires of behavior to obtain food. This is especially evident in insects, which exhibit a vast variety of feeding habits some of which have been conserved through evolution between insects and mammals. Insects prefer sweet compounds (Dethier, 1976; Gordesky-Gold et al., 2008; Masek and Scott, 2010) and reject bitter substances (Dethier, 1976; Sellier et al., 2011; Weiss et al., 2011). They modulate their food preference to compensate for the lack of salt (Trumper and Simpson, 1993; Simpson, 1994, 2006) and amino acids (Simpson and Abisgold, 1985; Simpson and White, 1990; Simpson et al., 2004; Mayntz et al., 2005; Ribeiro and Dickson, 2010; Vargas et al., 2010). Furthermore, feeding habits of insects have a strong ecological, economical, and medical impact, making them highly relevant for humans. Their impact can be negative and positive: while locusts and aphids are devastating agricultural pests and blood sucking makes mosquitoes vectors of deadly diseases, agriculture would be impossible without pollinating insects.

In this review we provide an overview of the neuronal and molecular mechanisms regulating insect feeding decisions. A comprehensive description of feeding behavior in blowflies was given by Dethier (1976). We focus on the feeding behavior of the adult *Drosophila melanogaster*, since the powerful molecular genetics of this model organism has provided the scientific community with many insights into the mechanisms of insect feeding behavior.

### Methods for measuring feeding and related behavior in insects

Feeding research relies on precise and robust measurements of food intake and feeding associated behaviors. In insects, especially in *D. melanogaster*, this is challenging due to the small size of the animals and the minute quantities of food they consume (Wong et al., 2008, 2009). Despite these challenges several methods have been developed to measure food intake, behavior associated with feeding or the activity of neurons involved in food consumption.

**Figure 1:**
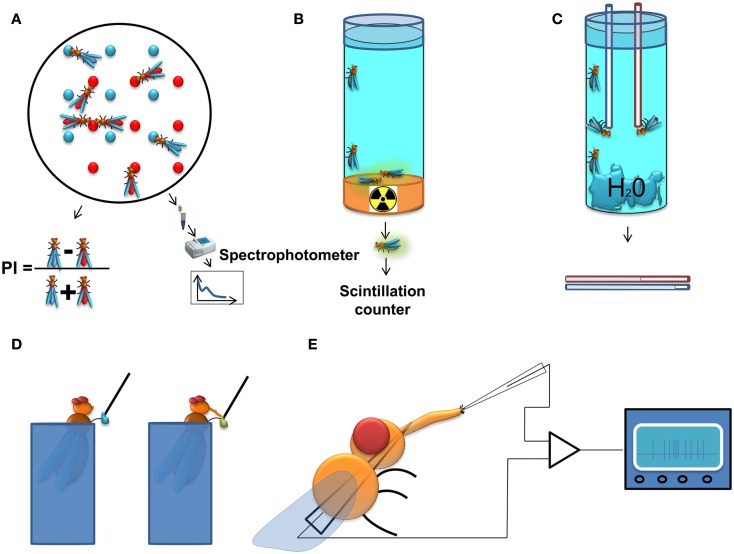
**Methods to study feeding behavior in *Drosophila***. **(A)** Two color food choice assay. Different sources of food are mixed with different dyes. The color of the abdomen of the flies is examined afterwards and a population preference index is calculated, or as an alternative approach, the dye content in the flies is determined using a spectrophotometer. **(B)** Radioactive food assay. Flies are kept on radioactive media and subsequently, the quantity of food consumed is measured with a scintillation counter. **(C)** CApillary FEeding assay. Several flies are kept in vials with a source of water and capillaries filled with food. The amount of food consumption is monitored by measuring the level of the meniscus in the capillaries. The assay can be used with either a single capillary to measure gross food intake, or with multiple capillaries with different food sources, thus providing quantitative information about the food preference of the flies. **(D)** Proboscis extension response (PER). In this assay the experimenter scores the probability of extension of the proboscis upon stimulation of the gustatory sensilla on the tarsi (depicted on the figure) or the labellum by a tastant solution. **(E)** Electrophysiological recording from gustatory sensillum. A fly is immobilized and a reference electrode is inserted through the thorax until it reaches the tip of the labellum. The recording electrode containing the tastant solution mixed with an electrolyte is positioned above the sensillum and the spiking activity of gustatory receptor neurons is registered and analyzed.

A classic approach is the two color choice assay to measure food preference (Tanimura et al., 1982; Ribeiro and Dickson, 2010; Dus et al., 2011). This assay (Figure 1A) is simple and allows high-throughput screening (up to 400 assays per person per week). For this test, flies are left to feed for a predetermined time from two different agarose food sources containing tastants mixed with different non-absorbable dyes. A qualitative readout can be achieved *post hoc* by visually scoring the color of the abdomen of the flies. To achieve a quantitative readout, the content of the digestive tract can be measured with the help of a spectrophotometer. Obviously, the use of one food source alone allows the quantification of food intake in a non-choice situation. A major disadvantage of this assay is that it does not allow dynamic monitoring of food intake across time as it normally relies on scoring dead flies, and does not take into account the food excreted by the flies.

An approach related to the colorimetric method is the use of a radioactively labeled food source for the acute measurement of food intake (Carvalho et al., 2006). This approach (Figure 1B) overcomes the background signal originating from the fly tissue in the spectrophotometric readout and is therefore more sensitive than the colorimetric assay. However, it allows only for the indirect comparison of food preferences (Vargas et al., 2010), and is therefore most useful for measurements of absolute food intake.

A method allowing dynamic measurement of food intake has recently been adapted for *Drosophila*. In the CApillary FEeding assay (CAFE; Ja et al., 2007) flies are allowed to eat from fine capillaries filled with liquid food and the consumed food is measured by assessing changes in the liquid levels within the calibrated capillary (Figure 1C). This assay can be used to directly measure food intake dynamically across time and has the sensitivity to discriminate individual sips of single fruit flies (Ja et al., 2007). It can also be used to measure food preference between various food sources using multiple capillaries (Lee et al., 2008; Sellier et al., 2011). Yet this method has several limitations: the flies are forced to eat in an upside-down position which could affect their feeding habits; the number of flies that are tested is rather small; and it is more laborious than the two color choice assay. The latter disadvantage could be overcome by automating the assay using a video-based imaging readout.

Among the methods for measuring behaviors associated to feeding, the Proboscis Extension Response (or reflex; PER) assay is one of the most widely used. Upon stimulation of the gustatory receptors on the labellum or the tarsae, hungry flies will extend their proboscis if the substance is palatable leading to the initiation of feeding (Figure 1D). Usually, the probability of the extension of proboscis is used as a quantitative measure in this assay (Dethier, 1976; Gordon and Scott, 2009; Chatterjee et al., 2010). This serves as a measure for the palatability of the tastant and the internal state of the animal, and is highly correlated with electrophysiological responses of the gustatory receptor neurons (GRNs; Dahanukar et al., 2007) as well as their calcium responses to tastants (Marella et al., 2006), but although proboscis extension always precedes a meal one can envisage that under certain circumstances it may not lead to food ingestion. Experimentally, the PER has several attractive qualities: it is reproducible between individual animals, can be performed on immobilized animals, and flies can be conditioned to extend their proboscis to stimuli for which they are initially unresponsive (DeJianne et al., 1985; Holliday and Hirsch, 1986; Chabaud et al., 2006). This last feature has served as basis for the use of the PER in studies on learning and memory (DeJianne et al., 1985; Holliday and Hirsch, 1986; Chabaud et al., 2006).

To achieve a mechanistic molecular and neuronal understanding of the regulation of feeding it is imperative to be able to survey the activity of the neurons crucially involved in the various aspects of feeding behavior (Figure 1E). Measuring the spiking activity of the GRNs is a well-established method (Hodgson et al., 1955) which provides a *bona fide* signal about the taste information that is transmitted from the sensory periphery to the central nervous system. This approach has been indispensable for the characterization of GRN responses to taste stimuli, and in revealing neuronal mechanisms underlying eating habits of insects and their modulation (Abisgold and Simpson, 1988; Simpson and Simpson, 1992; Chatterjee et al., 2010; Root et al., 2011). Recently, electrophysiological recordings have been expanded by the use of genetically encoded calcium indicators, which can be expressed specifically in neurons of interest, allowing the survey of larger populations of peripheral and central neurons (Marella et al., 2006; Fischler et al., 2007; Gordon and Scott, 2009; Root et al., 2011).

Given the complexity of feeding behavior several other methods can provide useful information about the behavioral and physiological changes associated with various internal states. Some examples are automated video tracking and fly activity monitoring (Lee and Park, 2004), the four field olfactometer assay (Meiners and Hilker, 1997; Faucher et al., 2006), biochemical examination of the hemolymph content as well as survival analyses.

## Feeding Decisions

When animals are faced with the option to feed, several decisions must be made: whether or not to eat, how much to eat, what to eat, and when to eat. Under certain assumptions, insects can be seen as systems trying to maintain homeostasis. From this point of view, feeding behavior serves to maintain nutritional homeostasis.

## To Eat or Not to Eat?

### The physiology of chemosensory systems in insects

Hungry animals need to locate external sources of nutrients and decide whether to ingest them in order to replenish internal resources and restore homeostasis. *Drosophila* possesses sophisticated sensory systems to detect the presence of nutrients, including the olfactory and gustatory systems, which have been extensively reviewed elsewhere (Scott, 2005; Hallem et al., 2006; Vosshall and Stocker, 2007; Benton, 2008; Vosshall, 2008; Masse et al., 2009; Montell, 2009; Tanimura et al., 2009; Touhara and Vosshall, 2009; Yarmolinsky et al., 2009; Isono and Morita, 2010). Here we will only briefly describe the key features of the gustatory and olfactory systems that are especially important to understand the regulation of feeding behavior.

The olfactory system of insects consists of olfactory sensilla, found on the antennae and maxillary palps. *Drosophila* has approximately 50 different types of olfactory receptor neuron (ORN), each of which expresses the same set of olfactory receptors or in exceptional cases receptors of the gustatory receptor gene family (Vosshall et al., 1999; Fishilevich and Vosshall, 2005). ORNs expressing the same receptor converge on the same glomeruli, dense neuropile structures in the antennal lobe (Vassar et al., 1994; Vosshall et al., 2000). Some of the ORNs express a novel gene family of glutamate Ionotropic receptors (IRs) instead of the olfactory receptors (Benton et al., 2009; Abuin et al., 2011). Unlike the olfactory receptors, several of these receptors are expressed in the same neuron (Benton et al., 2009), and at least for some of them it is clear that all of the neurons expressing the same receptor project to a single glomerulus in the antennal lobe (Benton et al., 2009). Within each glomerulus, ORNs form synapses with projection neurons (PNs) and a network of local interneurons. Approximately 180 PNs project to the mushroom bodies (MB), which are thought to be mainly involved in the formation of conditioned responses to odors (Margulies et al., 2005), and to the lateral horn (LH), which is thought to mainly mediate innate responses to odors (Masse et al., 2009).

The gustatory system of insects consists of gustatory sensilla, taste pegs, and internal taste organs. Both sensilla and taste pegs are found on the labellum, while the tarsae, and wings only harbor gustatory sensilla (Stocker, 1994). Interestingly, gustatory neurons on the *D. melanogaster* ovipositor have not yet been characterized, calling into question the existence of gustatory structures in this location. Unlike those in mammals, the gustatory sensory cells in insects are neurons (GRNs). There are four described types of GRNs in *Drosophila* (Falk et al., 1976): cells that respond to water (Fujishiro et al., 1984; Inoshita and Tanimura, 2006; Cameron et al., 2010), cells that respond to sweet substances (Fujishiro et al., 1984; Dahanukar et al., 2007), cells that respond to low concentrations of salt, and cells that respond to bitter substances (Meunier et al., 2003) and to high concentrations of salt (Fujishiro et al., 1984; Nakamura et al., 2002). The activation of the first three types of neurons promotes food consumption, while the activation of the last one triggers avoidance and suppresses feeding to prevent the animal from ingesting toxic substances (Marella et al., 2006). In addition to these taste categories, *Drosophila* is attracted to carbon dioxide in solution (carbonation) through a dedicated type of sensory neuron (Fischler et al., 2007). Recently it has been shown that some members of the IR family are expressed in the proboscis and could thus mediate some gustatory responses (Benton et al., 2009). Due to its labeled line architecture (Yarmolinsky et al., 2009), the gustatory system provides a very convenient regulatory point for feeding.

## Whether to Eat or Not and How Much to Eat

### Neuropeptides and neuromodulators as controllers of feeding

Feeding starts with a motivational drive that is determined by the current demands of the organism. In *Drosophila*, as in many other animals, this demand can be mediated by neuropeptides within the nervous system (Nässel and Winther, 2010). Within the scope of this review we will focus on the following neuropeptides: Hugin, Neuropeptide F (NPF), short Neuropeptide F (sNPF), Insulin-Like Peptides, and Leucokinin.

The *hugin* gene encodes a neuropeptide homologous to mammalian Neuromedin U (Melcher et al., 2006), which is expressed in the suboesophageal ganglion of adult and larval *Drosophila* (Bader et al., 2007a,b). It was identified as a gene that is upregulated in *pumpless* and *klumpfuss*, mutants with deficits in larval feeding behavior (Melcher and Pankratz, 2005). The expression of *hugin* is suppressed in *Drosophila* larvae by both starvation and yeast deprivation. Overexpression of *hugin* suppresses feeding in the larva, while inhibition of *hugin* expressing neurons with tetanus toxin reduces the latency to initiate feeding in adult flies. Therefore *hugin* expressing neurons (and to a certain extent *hugin* itself) seem to be responsible for the control of the initiation of feeding as it suppresses immediate feeding responses and is downregulated by starvation and amino acid deprivation (Melcher et al., 2007).

**Figure 2:**
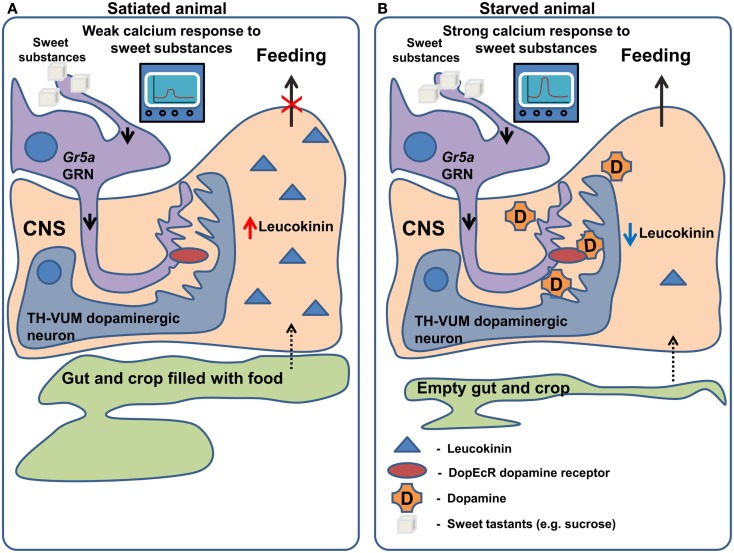
**Regulation of gustatory processing and feeding behavior by starvation**. **(A)** In satiated animals, leucokinin is released in response to the filling of the crop and gut occurring after feeding. Leucokinin affects unknown populations of neurons in the nervous system via leucokinin receptors resulting in the termination of feeding. In the same animals TH-VUM dopaminergic neurons are less active and *Gr5a* expressing GRNs produce a weak response to “sweet” compounds. **(B)** In starved animals, the spiking activity of TH-VUM neurons is increased leading to dopamine release on the *Gr5a* expressing GRNs. In these GRNs dopamine binds to the DopEcR receptor causing increase in the calcium response to “sweet” compounds. As the crop and gut are empty leucokinin release is inhibited and feeding termination does not occur.

Another neuropeptide, leucokinin, which is a potential homolog of mammalian Tachykinin, may signal the amount of food in the foregut and thus controls the termination of the meal (Figure 2). This is apparent from the behavioral phenotypes of both *leucokinin* and *leucokinin receptor* mutants: the mutant animals increase the amount of food they consume per meal and, as a compensation, increase the inter-meal interval, keeping the caloric intake constant (Al-Anzi et al., 2010). The same behavioral phenotype can be observed in animals with ablated *leucokinin* expressing neurons. Neuronal leucokinin is responsible for this phenotype since the phenotype can be rescued by the pan-neuronal expression of either the peptide or its receptor. Furthermore, the effect of leucokinin appears to be independent of hugin and npf neurons since their ablation does not affect meal size (Al-Anzi et al., 2010). In short, leucokinin appears to mediate the decision to stop feeding.

**Figure 3:**
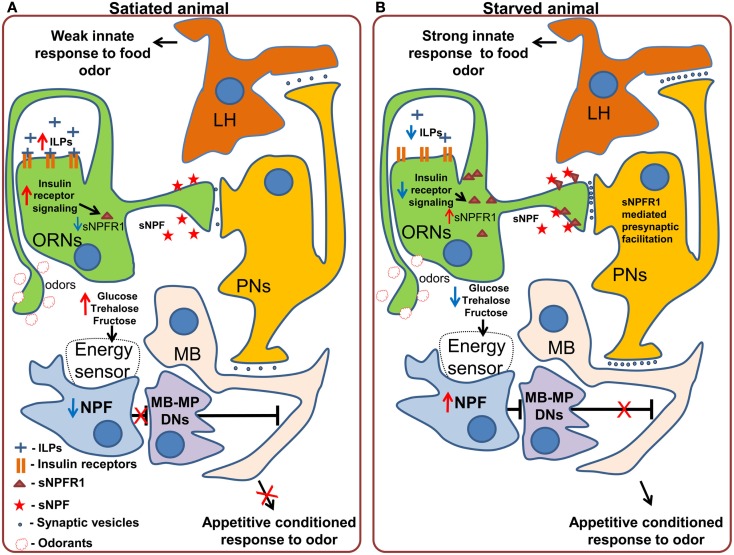
**Effects of starvation on olfactory processing in *Drosophila***. **(A)** In satiated animals, innate responses to food odors, which are probably mediated by the lateral horn (LH) are weak. A subpopulation of dopaminergic neurons (MB-MP DNs) projecting to the mushroom body (MB) suppresses the output of the mushroom body via tonic release of dopamine. While these neurons are active, retrieval of appetitive conditioned responses to odors does not occur. **(B)** In starved animals, the inhibition of Insulin-like receptor (InR) signaling in the olfactory receptor neurons (ORNs) stimulates the synthesis and incorporation of Short Neuropeptide F Receptor type 1 (sNPFR1) into the membrane of these neurons. sNPFR1 mediates presynaptic facilitation of the ORN response to odors, increasing the activity of projection neurons (PNs), and enhancing the innate response to attractive food odors, presumably mediated by the lateral horn neurons as well as conditioned responses to odors by the mushroom body neurons. At the same time the decrease in the hemolymph concentration of glucose and trehalose is detected by an internal energy sensor (which may or may not be directly connected to NPF neurons) which in turn activates Neuropeptide F expressing neurons. Activation of the NPF receptor 1 leads to the inhibition of MB-MP dopaminergic neurons and thus the release of the output of the mushroom body from tonic dopaminergic inhibition, allowing the retrieval of conditioned appetitive responses.

Neuropeptide F, is an ortholog of mammalian Neuropeptide Y and shares its involvement in the regulation of food intake (Tatemoto et al., 1982; Wu et al., 2005a,b; Krashes et al., 2009; Nässel and Wegener, 2011). NPF should not be confused with short Neuropeptide F, which performs different functions and which we will discuss separately (Nässel and Wegener, 2011). In *Drosophila* larvae, NPF receptor 1 (NPFR1) activation promotes feeding on noxious food as well as solid (unattractive) food, mimicking the effect of starvation (Wu et al., 2005a,b). These effects are partially mediated by the suppression of the RPS6-p70-protein kinase (S6K) and by Insulin-like receptor (InR) signaling in NPFR1 neurons. In the neurosecretory neurons that produce ILP (Insulin-like peptide), the same S6K cascade affects the intake of both liquid and solid food and is mediated by changes in the release of ILP2 and ILP4 (Wu et al., 2005a). NPF has also been shown to be necessary for the recall of olfactory appetitive memory in adult flies through action on so called MB-MP dopaminergic neurons, which send their efferents to the mushroom body (Krashes et al., 2009). In satiated animals, the NPF neurons are silent and the output of the mushroom body is inhibited by the MB-MP neurons (Figure 3A). In starved animals NPF neurons are activated, leading to inhibition of the dopaminergic MB-MP neurons. The opening of the inhibitory gate from the MBs allows for the recall of the appetitive conditioned responses to odors and subsequent attraction toward presumptive appetitive food sources (Figure 3B).

Another peptide that conveys information about the state of internal energy resources is sNPF. Initial experiments showed that pan-neuronal overexpression of *snpf* stimulates feeding in flies, while downregulation of *snpf* using neuron-specific RNAi suppresses feeding (Lee et al., 2004). Furthermore, overexpression of *snpf* not only affected feeding behavior, but also growth, as evidenced by the induced changes in fly size, suggesting that the observed phenotypes might be at least partially due to changes in fly metabolism and growth signals (Lee et al., 2004). Recently, Root et al. (2011) uncovered an elegant mechanism by which sNPF acts to modulate neuronal circuits relevant for feeding. Signaling through the sNPF receptor, sNPFR1, in ORNs mediates an increase in olfactory sensitivity to food odors (cider vinegar) in starved flies. Using video tracking based analysis of foraging the authors demonstrated that after starvation, flies became more sensitive to the vinegar odor. This behavioral change is implemented at the neuronal level by an increased sensitivity of the ORNs carrying information about the appetitive odor. Overexpression of sNPFR1 and sNPF in Or42b expressing sensory neurons was sufficient to mimic starvation. Interestingly, the increased sensory responses to vinegar upon starvation are due to the upregulation of the expression of sNPFR1, without any changes at the level of the peptide itself. This upregulation is triggered by suppression of insulin signaling in the ORNs, and is both necessary and sufficient to mimic the effect of starvation on olfactory perception. In this case, while not acting on feeding itself, but on foraging, the modulation occurs mainly at the peripheral level (Figure 3) and is mediated by the enhancement of olfactory attractiveness of a food odor – vinegar, a reliable cue for *Drosophila*’s favorite meal – rotten fruit.

While the examples mentioned above describe effects of neuropeptides on the olfactory system, very recently two groups (Inagaki et al., 2012; Marella et al., 2012) independently discovered that the neuromodulator dopamine mediates increased GRN sensitivity in hungry flies (Figure 2). The findings of Inagaki et al. (2012) rely on the use of a new method which allows the mapping of sites of action of dopamine. They demonstrate that during starvation dopamine signaling is increased in Gr5a sugar sensing GRNs, leading to an increased probability of proboscis extension, a key step in food intake. The effects of dopamine on sugar sensing neurons are in turn mediated specifically by the DopEcR dopamine receptor, a receptor whose physiological function had previously remained elusive. A second group (Marella et al., 2012) identified a dopaminergic neuron named TH-VUM, which projects to the suboesophageal ganglion (the site of the brain to which GRNs project). This neuron is activated by starvation increasing the probability of the extension of the proboscis to sucrose. In fact, activation of the TH-VUM in isolation using TRPA1 induces proboscis extension, while silencing of this neuron inhibits it (Marella et al., 2012). These studies demonstrate that dopamine is a key player in enhancing gustatory sensitivity toward sugars upon starvation, shedding light on the longstanding question of how hunger facilitates the extension of the proboscis.

In the picture which emerges, starvation causes changes in levels of specific neuropeptides and neuromodulators affecting feeding decisions via central and peripheral mechanisms. Acting on the periphery, they enhance innate responses to attractive odors and sugars (InR > sNPFR1 > presynaptic facilitation in the ORNs and TH-VUM > DopEcR > increased Calcium responses of the GRNs) while their action in the brain (NPF > MB-MP neurons > MB; Figure 3), alters the acquired attractiveness to odors in a metabolic state dependent way (Figures 2 and 3).

## Whether to Eat and What to Eat

### Internal state sensors

To ensure feeding homeostasis, internal sensor mechanisms must be present that signal the lack or excess of internal nutritional resources. Internal sensors are necessary not only to initiate feeding *per se*, but also to assist in the selection of the optimal food source to compensate for the lack of specific nutrients. In insects, there is behavioral evidence for the existence of internal energy sensors (Burke and Waddell, 2011; Dus et al., 2011; Fujita and Tanimura, 2011) as well as protein/essential amino acid sensors (Simpson and Abisgold, 1985; Lee et al., 2008; Ribeiro and Dickson, 2010; Vargas et al., 2010). This extends earlier behavioral findings in rats demonstrating the existence of internal energy sensors (Sclafani and Nissenbaum, 1988). The molecular and neuronal substrates of the internal sensors are currently under intense investigation and the first details of their functioning are the main focus of this review. In general these sensors could directly enable the neurons to sense levels of nutrients, or could act on neurons via surrogate signals (hormones) secreted by nutrient sensing cells outside the nervous system.

### Experimental evidence for the existence of energy sensors

While neuropeptides mediate changes in behavior by modifying information processing in the nervous system, the question remains as to how the neuropeptide releasing neurons detect the internal nutritional state of the animal. Recently, three groups have independently produced behavioral evidence for an internal energy sensor in *Drosophila*. Similar mechanisms are thought to exist in mammals (Sclafani and Nissenbaum, 1988; de Araujo et al., 2008; Oliveira-Maia et al., 2011) and have been recently reviewed (De Araujo, 2011). An early line of evidence for the existence of an internal energy sensor comes from the work of Sclafani and Nissenbaum (1988). Their work demonstrated that pairing of flavored water with intra-gastric infusions of hydrolyzed starch in rats led to strong and robust flavor preference in favor of the starch-paired flavor. This work demonstrated that the caloric value *per se* can be rewarding, and is capable of modifying behavior. Recently, It has been demonstrated that flies too can be conditioned by the caloric value of the food (Burke and Waddell, 2011; Dus et al., 2011; Fujita and Tanimura, 2011). This is shown by the fact that flies can be conditioned using sorbitol, an alcohol that can be used by flies as an energy source, but to which they do not show any gustatory responses (Fujita and Tanimura, 2011). In contrast, if flies are conditioned with sweet but non-caloric food (arabinose) the memory trace is weak and unstable. This memory trace can, however, be stabilized if attractive but non-metabolizable sugar (arabinose) is mixed with tasteless but energy-rich substance (sorbitol; Burke and Waddell, 2011). These experiments demonstrate the existence of an internal energy sensor, working in parallel with gustatory perception that is crucial for memory formation and stabilization.

These observations are supported by a second set of experiments showing that starved “taste-blind” fruit flies prefer sucrose over a non-caloric alternative. Several genes have been shown to be crucial for the gustatory detection of sucrose. Among these are the trehalose receptor Gr5a (Dahanukar et al., 2001) and Gr64a, a receptor for maltose, sucrose, and glucose (Jiao et al., 2007). Both of these receptors are expressed in sugar sensitive GRNs (Dahanukar et al., 2007). The other gene in which mutations lead to a severe taste deficit is *Pox neuro*, which encodes a transcription factor (Awasaki and Kimura, 1997). In *Pox neuro* mutant flies all chemosensory sensilla are transformed into mechanosensory organs, leading to a loss of gustatory perception. Dus et al. (2011) tested several “sugar – blind” flies, either *Gr5a* and *Gr64a* double mutants or *Pox neuro* mutants, and found that neither of the taste-blind flies showed a significant PER when presented with 100 mM sucrose before or after starvation. However, in the two color choice assay they ate significantly more of the sucrose-containing agar gel than the agar gel alone. Furthermore, when given the choice between two sugars that are both perceived as sweet by the wild type flies, but differ in their nutritional content (non-metabolizable sucralose or l-glucose and metabolizable sucrose or d-glucose), starved *Gr5a* and *Gr64a* double mutant flies ate significantly more of the metabolizable sugars (Dus et al., 2011). This preference for calorie-rich food was correlated with the depletion of glycogen reserves and decreased hemolymph levels of glucose and trehalose. These findings suggest that flies are capable of postingestive identification of calorie-rich food through a putative internal energy sensor independent from the characterized gustatory “sweet” receptors (*Gr64a* and *Gr5a*) or other chemoreceptors on *poxn* positive taste sensilla.

These studies provide converging evidence for the existence of a behaviorally relevant energy sensing mechanism in *Drosophila*, but multiple questions still remain. Are energy levels sensed by the nervous system directly? Which molecular machinery is used to sense energy in the nervous system? Which cells act as energy sensors?

Current research is starting to answer these questions. Following up previous observations (Thorne and Amrein, 2008; Park and Kwon, 2011), a gustatory receptor (Gr43a) has been identified as being expressed not only in the gustatory organs but also in the digestive tract, uterus, and most importantly in the central brain of *Drosophila* where it acts as an internal energy sensor (Miyamoto et al., 2012). In a series of elegant experiments the authors demonstrated that Gr43a is necessary for fructose sensing and that, unexpectedly, fructose levels in the hemolymph constitute a reliable postingestive signal to estimate the energy content of a meal. Only three to four neurons in the dorsolateral protocerebrum express Gr43a receptors and show robust Calcium responses to fructose within the physiological range of concentrations. The activity of these fructose sensing neurons is likely to play an important role in mediating the metabolic effect of carbohydrate ingestion on feeding behaviour and short term memory (Miyamoto et al., 2012).

These findings do not contradict the possibility that in *Drosophila* either the NPF neurons themselves (as described above) can act as energy sensors, or that a different subset of neurons or tissues act as energy sensors and indirectly exert their function via NPF release.

### Experimental evidence for the existence of internal protein/amino acid sensors

Research on the neuronal basis of food intake and energy expenditure has largely concentrated on energy-rich bulk food intake and energy homeostasis, largely ignoring other types of nutrients such as proteins. This stands in contrast to the substantial body of evidence that has accumulated over the last 30 years suggesting that different species of animals, both vertebrates and invertebrates, are capable of selecting food sources that optimize not only the gross energy intake, but also the intake of macronutrients such as amino acids, salts, and sterols (Trumper and Simpson, 1993; Behmer and Joern, 1994; Simpson, 1994, 2006; Behmer et al., 1999; Behmer, 2009). A comprehensive review of the different behavioral adaptations to imbalanced diets in insects has recently been discussed in detail elsewhere (Behmer, 2009). We would like to focus on the emerging neuronal and molecular mechanisms underlying the regulation of protein intake.

Locusts are herbivores that change their food intake upon exposure to a low protein diet. They increase total food consumption by decreasing inter-meal interval without changing the size of individual meals (Simpson and Abisgold, 1985). This seminal discovery spurred a large body of work which has made important contributions to our current understanding of the physiological and neuronal mechanisms underlying protein homeostasis. In locusts kept on a low protein diet, hemolymph osmolality and amino acid concentrations decrease, followed by an increase in food intake (Abisgold and Simpson, 1987). Accordingly, injecting of amino acids directly into the hemolymph or raising hemolymph osmolality partially reverses the increase in food consumption. Furthermore, simultaneous increase of hemolymph osmolality and amino acid concentrations resulted in even larger inter-meal intervals, suggesting that both physiological parameters independently influence the increase in food intake. The same authors ruled out feedback from stretch receptors as being involved in regulating this behavior (Abisgold and Simpson, 1987). These results suggested the existence of an internal amino acid sensor controlling feeding behavior in locusts.

**Figure 4:**
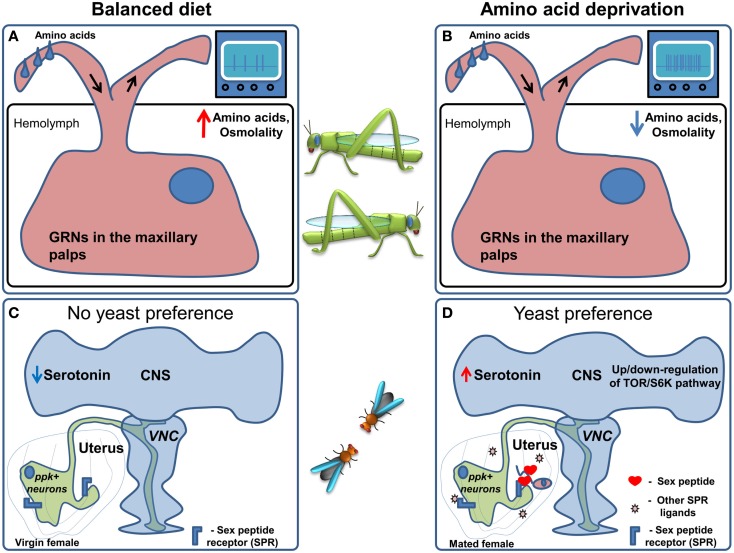
**Regulation of amino acid preference**. **(A)** When locusts are kept on a balanced diet the sensitivity of amino acid responsive GRNs in the maxillary palps is moderate. **(B)** Upon amino acid deprivation hemolymph amino acid concentration and osmolality are decreased. This leads to an increase in the sensitivity of amino acid responsive GRNs followed by an increase in food consumption. **(C)** In *Drosophila* kept on a balanced diet *ppk*^+^ neurons are active, causing flies to prefer sucrose. **(D)** When flies are deprived of amino acids, it is conceivable that, TOR/S6K signaling in neurons is altered presumably indicating an internal amino acid deficiency and serotonin levels in the brain are thought to increase. Furthermore, upon mating Sex Peptide, as well as other ligands of the Sex Peptide receptor, are transferred to the uterus causing the inhibition of the *ppk*^+^ neurons which project to the Ventral Nerve Cord (VNC) and the brain. These changes lead to an increase in yeast (amino acid rich food) preference.

Interestingly, in locusts the sensitivity of maxillary palp GRNs (Figures 4A,B) is correlated with the increase in food intake seen in response to the low protein diet: the sensitivity of the GRNs to leucine and a mixture of 10 amino acids increased, with no apparent change in the sensitivity to sucrose (Abisgold and Simpson, 1988). Importantly, injection of amino acids into the hemolymph reversed the change in receptor sensitivity to pre-deprivation levels (Abisgold and Simpson, 1988). The effects on the sensitivity of GRNs were not mediated by a top-down effect from the central nervous system, since transection of the maxillary nerve did not affect the changes in sensitivity, which could be reversed by injection of amino acids directly in to the isolated maxillary palp (Simpson and Simpson, 1992). In locusts the current hypothesis is that the amino acid sensor is likely to be located in the GRNs themselves, and that the increased consumption of proteins is largely determined by elevated sensitivity of GRNs to amino acids. This stands in contrast to vertebrates, where protein homeostasis is thought to rely on amino acid sensing in the brain (Hao et al., 2005; Maurin et al., 2005; Gietzen et al., 2007). Following these discoveries, further research suggested that protein intake is tightly regulated on a behavioral level in many different species (Raubenheimer and Simpson, 1997), leading to the development of a unifying methodological and theoretical framework which was termed “nutritional geometry” (Raubenheimer and Simpson, 1993; Lee, 2006).

Despite these important contributions the locust as a model organism is not very well suited for the dissection of molecular and neuronal mechanisms. Recently *D. melanogaster* has been shown to be able to tightly regulate protein intake (Ribeiro and Dickson, 2010; Vargas et al., 2010) to achieve maximal reproductive success (Lee et al., 2008), opening up the possibility to use its sophisticated neurogenetic toolkit to study this important nutritional process (Figures 4C,D).

When given the choice between protein rich food (yeast) and carbohydrate-rich food (sucrose) using the two color choice assay, fruit fly males and females differ dramatically in their response to protein deprivation (Ribeiro and Dickson, 2010). Flies of both sexes normally prefer sucrose solutions over yeast. Males switch their preference to yeast after 10 days of protein deprivation, and while virgin females behave much like males, females switch their preference to protein rich food much faster after mating, i.e., after 3 days of yeast deprivation. This behavioral change is at least partially mediated by the Sex peptide, a short peptide contained in the seminal fluid of males that is injected into the female during copulation and is thought to act on the Sex peptide receptor (Fox et al., 1959; Kubli, 2003; Yapici et al., 2008). Furthermore, Sex peptide receptor acts in neurons in the female genital tract called *pickpocket*^+^ *(ppk)*^+^ neurons (Häsemeyer et al., 2009; Yang et al., 2009) sending projections to multiple brain areas (Häsemeyer et al., 2009; Rezával et al., 2012) to modulate food preference (Ribeiro and Dickson, 2010). In addition to *ppk*^+^ neurons, Target of Rapamycin/S6K (TOR/S6K) signaling in the nervous system and serotonin are likely regulators of this nutritional decision. In fact, modulation of the TOR pathway or the activity of one of its downstream targets – S6K – in the nervous system of males or virgin flies causes a clear preference for yeast over sucrose (Ribeiro and Dickson, 2010; Vargas et al., 2010). Given the fact that the TOR/S6K pathway is best known as a cellular nutrient sensing pathway reporting the lack of amino acids (Wullschleger et al., 2006; Liao et al., 2008), and has been shown to regulate feeding behavior in vertebrates (Cota et al., 2006), it is very attractive to speculate that this pathway could act as a neuronal nutrient sensor, underlying changes in feeding decisions upon ingestion of imbalanced diets.

Despite these encouraging first insights into possible molecular mechanisms regulating feeding decisions upon protein deprivation, many questions still remain open. It will be important to determine whether TOR/S6K signaling indeed acts as a nutrient sensor in the nervous system and, if so, in which neurons it acts. A further important question is how changes in nutrient sensing, be it mediated by TOR/S6K activity and serotonin or by another mechanism, are translated into neuronal activity and ultimately changes in feeding decisions. To answer these questions, the identification of further molecular players mediating this homeostatic nutritional behavior will be essential. Regarding the modes of action, multiple hypotheses of how internal nutrient sensing leads to changes in nutrient choice can be envisaged. It is possible that, similar to what has been proposed in locusts, nutrient sensing in *Drosophila* acts at the level of peripheral chemosensory neurons. An obstacle to gaining further insight into this aspect of protein homeostasis is that, in contrast to locusts and many other insects, *Drosophila* has not been shown to have functional GRNs sensitive to amino acids. The recent demonstration, however, that fruit flies can taste amino acids (Toshima and Tanimura, 2012) is an important step toward the identification of *Drosophila* amino acid receptor neurons. Due to the specialization for yeast feeding, *Drosophila* is furthermore likely to have evolved to use yeast metabolic products, such as carbon dioxide (Fischler et al., 2007) or glycerol (Koseki et al., 2005; Wisotsky et al., 2011), as proxies signaling the availability of amino acids. Further insights into nutrient choice in this genetically tractable organism will therefore require a better understanding of the chemosensory basis for detection of amino acid rich food. A different hypothesis is that a postingestive mechanism to detect the lack of amino acids in the diet affects nutrient decisions through the modulation of higher brain centers, as has been described in vertebrates (Gietzen et al., 2007). Ultimately, a combination of peripheral and central modulation, as is the case for energy homeostasis, is most likely to occur. In any case, further understanding of the molecular basis for nutrient choice in *Drosophila* will rely on the identification of more molecular and neuronal players and a better electrophysiological, cellular, nutritional, and behavioral understanding of how they act within the nervous system to modify feeding decisions.

## When to Eat

### The influence of circadian rhythms and sleep on feeding decisions

To achieve an optimal expenditure of organismal resources and to maximize fitness (Xu et al., 2011), physiological processes are orchestrated by the circadian clock machinery (Sehgal, 1995). It is therefore not surprising that the same holds true for feeding behavior (Krishnan et al., 2008; Tanoue et al., 2008; Xu et al., 2008; Chatterjee and Hardin, 2010).

**Figure 5:**
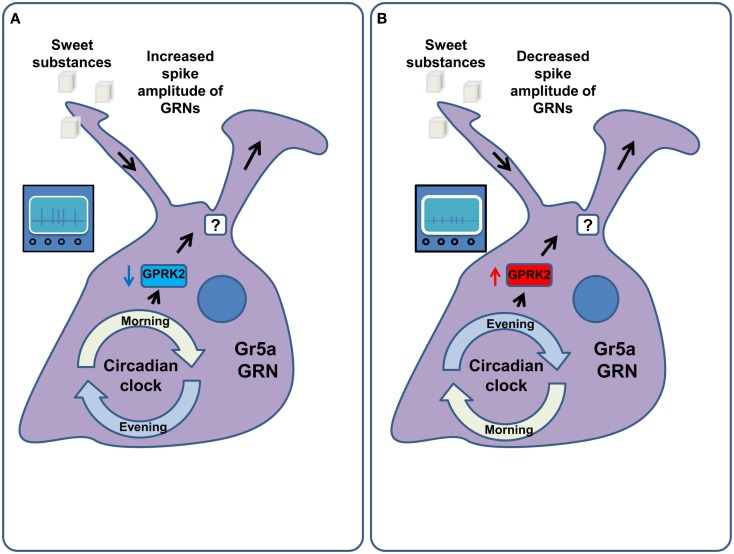
**Circadian regulation of GRN sensitivity**. **(A)** In the morning the molecular machinery of the circadian clock down-regulates the activity of the G-Protein coupled receptor regulatory kinase 2 (GPRK2) in the Gr5a gustatory receptor neurons. This amplifies the responsiveness of these GRNs to sucrose via an increase in their firing rate as well as the width and amplitude of their action potentials. As a result flies consume more food in the morning. **(B)** In the evening and at night the activity of GPRK2 is increased, leading to a decrease in the sensitivity of Gr5a GRNs to sucrose. This is mediated by decreasing the spike width and amplitude of these GRNs. As a result flies consume less food in the evening.

In *Drosophila*, the sensitivity of GRNs to tastants does not remain constant throughout the day (Chatterjee et al., 2010) with a maximum sensitivity in the morning (Chatterjee and Hardin, 2010). This phenomenon is mediated by the G-Protein coupled receptor regulatory kinase 2 (GPRK2), which, in turn, is regulated by the circadian molecular clock machinery in the GRNs themselves (Figure 5). The food intake of flies follows the sensitivity of the receptor neurons, with peak food intake in the morning. Diurnal variations of sensitivity in *Drosophila* ORNs have also been described. They are also mediated by GPRK2 (Tanoue et al., 2008), but display the opposite regulation to that observed in the gustatory system, such that the peak of olfactory sensitivity is at night, when gustatory sensitivity is minimal. Surprisingly, the changes in sensitivity were proposed to be mediated not only by the alteration of the firing rate in response to stimuli, but also by changes in the amplitude and duration of the action potential generated in the GRNs and ORNs (Krishnan et al., 2008; Chatterjee et al., 2010). Confirmation of these observations and the investigation of the exact nature of this phenomenon remain to be uncovered by future studies and will require either calcium imaging or ideally patch clamp recordings as opposed to the extracellular tip recordings used in these studies. Furthermore, circadian clock components in peripheral tissues also regulate feeding. Interfering with components of the circadian clock in the fat body, for example, disrupts the circadian pattern of feeding while increasing food consumption and decreasing the levels of glycogen and resistance to starvation (Xu et al., 2008).

Another mechanism that has been proposed to mediate changes in food intake across the diurnal circle is mediated by the protein Takeout. *takeout* has strong sequence homology to the Juvenile Hormone (JH) binding protein genes, whose products are involved in the transport of the lipophilic JH to its tissue targets. Takeout was isolated as a protein whose expression is strongly regulated by the circadian rhythm and was shown to affect feeding: *takeout* mutant flies overeat when food is available *ad libitum* (Sarov-Blat et al., 2000; Meunier et al., 2007). In addition, *takeout* mutants do not show a decrease in GRN sensitivity to glucose after feeding, suggesting that the mechanism of overeating is caused by a lack in modulation of the sensitivity of the peripheral GRNs (Meunier et al., 2007). These results provide further evidence for the importance of peripheral chemosensory modulation in the regulation of feeding.

As feeding and sleeping are mutually exclusive behaviors, any description of the regulation of food intake would be incomplete without a discussion of its coordination with sleep. In vertebrates, long term sleep deprivation stimulates appetite (Rechtschaffen and Bergmann, 2002) while starvation leads to a decrease in sleep (MacFadyen et al., 1973). Mechanistically these behaviors are coordinated by the orexin/hypocretin system, which controls both food intake and wakefulness (De Lecea et al., 1998; Sakurai et al., 1998) and which is regulated by blood amino acid and glucose levels (Burdakov et al., 2005, 2006; Frederick-Duus et al., 2007; Karnani et al., 2011). Fruit flies also decrease the time they sleep when they are starved (Lee and Park, 2004; Keene et al., 2010), and yeast feeding can shorten or terminate the sleep of flies (Catterson et al., 2010). As the effect of starvation on sleep is mediated by nutrient deprivation it is likely to involve internal energy sensing. Accordingly feeding sucralose, a non-nutritious sweet compound, to starved flies does not lead to an increase in sleep (Keene et al., 2010). Furthermore, this starvation-induced sleep alteration is mediated by *clock* and *cycle* in the dorsally located population of the *clock* expressing neurons (Keene et al., 2010), opening a window to a better understanding of how these essential, but mutually exclusive behaviors are coordinated.

## Concluding Remarks, Open Questions, and Future Directions

Despite the recent increase in knowledge of the molecular and neuronal components as well as the mechanisms controlling feeding decisions in *Drosophila*, important questions still remain to be answered.

One of the main open questions is the exact nature of the nutrient sensing mechanisms. We are just starting to identify the molecular machinery allowing the nervous system to detect the lack of energy available to the fly, the neurons in which this machinery acts, and to which extent nutrient sensing happens within the nervous system. It will be interesting to differentiate between two possibilities. One in which nutrient sensing happens centrally in a small set of neurons or in a peripheral organ that systemically regulates the activity of all neurons involved in feeding decisions. The other possibility would be that all or a majority of neurons are able to sense the lack of specific nutrients, and use this information to specifically modify their mode of action to modulate feeding.

The emerging picture of how feeding decisions are modulated in *Drosophila* is that upon changes in nutrient state, both peripheral chemosensory and central neurons change their firing properties to elicit a change in feeding behavior (Figures 2 and 3). Interestingly, gustatory receptors are also expressed outside of the taste organs, for example in the midgut and in the uterus (Miyamoto et al., 2012). The function of these “ectopic” receptors is not defined; however it is interesting to speculate that they may also be involved in the evaluation of the internal state of the animal.

As the molecular and neuronal mechanisms underlying nutrient sensing, and the ways in which they elicit changes in feeding behavior, are better understood, the challenge will be to integrate this knowledge into a systems-level framework of how changes in neuronal output are translated into a whole-animal response to ensure homeostasis. This will also require an understanding of how the different systems ensuring the homeostasis of energy, protein, and other nutrients interact at the behavioral, neuronal, and molecular level to maximize survival chances and reproduction.

This systems-level understanding will rely on expanding the repertoire of behavioral assays used to study feeding (Figure 1) in order to be able to capture, quantitatively and dynamically, the full complexity of feeding and associated fly behaviors. Video tracking and automatic analysis of behavior, which arose from the intersection between machine vision and ethology (Branson et al., 2009; Fontaine et al., 2009; Straw et al., 2011), might fulfill these requirements, particularly if they are expanded by methods for online monitoring of food consumption. Complementary monitoring of neuronal activity during behavior will be important to understand how neuronal computations lead to feeding decisions to ensure homeostasis.

Ultimately, the combination of the identification of molecular and neuronal mechanisms and fine behavioral data in genetically and nutritionally manipulated animals, together with associated changes in neuronal dynamics, will allow us to build an understanding of how the animals make feeding decisions allowing them to maintain the stability of the internal milieu.

## Conflict of Interest Statement

The authors declare that the research was conducted in the absence of any commercial or financial relationships that could be construed as a potential conflict of interest.
